# The effects of intracisternal enzyme replacement versus sham treatment on central neuropathology in preclinical canine fucosidosis

**DOI:** 10.1186/s13023-015-0357-z

**Published:** 2015-11-04

**Authors:** Gauthami Sudhamayee Kondagari, Jessica Louise Fletcher, Rachel Cruz, Peter Williamson, John J. Hopwood, Rosanne Maree Taylor

**Affiliations:** Faculty of Veterinary Science, University of Sydney, Camperdown, NSW 2006 Australia; Lysosomal Diseases Research Unit, South Australian Health and Medical Research Institute, Adelaide, SA 5000 Australia

**Keywords:** Fucosidosis, Intracisternal enzyme replacement therapy, Neuroinflammatory markers, Canine model, Neurodegeneration, CNS

## Abstract

**Background:**

Fucosidosis results from lack of α-L-fucosidase activity, with accumulation of fucose-linked substrates in the nervous system and viscera leading to progressive motor and mental deterioration, and death. The naturally occurring dog model of fucosidosis was used to evaluate the neuropathological responses to partial enzyme replacement, and substrate reduction in early disease following treatment with recombinant canine α-L-fucosidase delivered through cerebrospinal fluid.

**Methods:**

Neuropathology in both treated (*n* = 3) and untreated fucosidosis-affected (*n* = 3) animals was evaluated with immunohistochemistry, image analysis, manual quantification and gene expression analysis and compared with unaffected age-matched controls (*n* = 3) in an extension of our previous biochemical report on the same cohort. Data were analyzed by ANOVA.

**Results:**

Quantification demonstrated a consistent trend to reduction in vacuolation, pyramidal neuron loss, astrocytosis, microgliosis, perivascular storage, apoptosis, oligodendrocyte loss, and hypomyelination throughout the central nervous system of enzyme treated animals compared to placebo-treated, age-matched affected controls. Key lesions including lysosomal expansion in neurons of deep cortex, astrocytosis in cerebral cortex and medulla, and increased lysosomal membrane associated protein-1 (LAMP-1) gene expression were ameliorated in treated animals. There was no change in spheroid formation and loss of Purkinje cells, but Purkinje cell vulnerability to apoptosis was reduced with treatment.

**Conclusions:**

Despite reduced severity of fucosidosis neuropathology with partial enzyme replacement, more complete and sustained biochemical correction is required to halt neuropathological processes in this large animal model of lysosomal storage disease.

**Electronic supplementary material:**

The online version of this article (doi:10.1186/s13023-015-0357-z) contains supplementary material, which is available to authorized users.

## Background

Canine fucosidosis is an animal model for the homologous human lysosomal storage disorder (LSD) resulting from the loss of functional α-L-fucosidase enzyme activity. It is the only preserved animal model of fucosidosis (OMIM#23000), and has been invaluable in testing treatment strategies, such as haematopoietic cell transplantation (HCT) [1]. Characteristic features of fucosidosis neuropathology including with accumulation of fucosyl glycoasparagines, lysosomal expansion, hypomyelination, gliosis, neuronal loss, axonal dystrophy and apoptosis are present in the canine model by 8 weeks of age; 8–10 months before the onset of motor and mental deficits [2, 3]. Consistent signs of ataxia, hypermetria and behavioral changes commence at 12–18 months and at this stage, there is widespread vacuolation in neurons and glia across the brain, and a 16 % decrease in myelin density accompanied by an average 38 and 54 % loss of oligodendrocytes in the cerebellum and corpus callosum [2, 4]. By 24 months, affected animals demonstrate severe gait deficits, spontaneous nystagmus and loss of menace response, with an average of 76 % fewer Purkinje cells and 59 % fewer pyramidal neurons compared to normal age-matched adults [3].

In canine fucosidosis, HCT increased α-L-fucosidase activity to more than 10 % of normal in the nervous system 6 months after engraftment, ameliorated neuropathological lesions and there was a substantial, life-long reduction in clinical signs [1, 5, 6]. HCT is an option for fucosidosis-affected infants if diagnosed soon after birth, but requires a compatible donor and entails substantial risks with limited benefit in severe disease or in late-onset cases [7, 8]. There is a compelling need for a safe and successful treatment for fucosidosis, which due to the neurologic involvement requires the delivery of therapeutic agents across the blood-brain barrier. This may be achieved by direct delivery of replacement enzyme into the cerebrospinal fluid (CSF) via intrathecal or intracisternal infusion. In canine fucosidosis, low-dose, monthly intracisternal enzyme replacement therapy (ERT), increased enzyme activity by 2–72 % in the spinal cord and most areas of the brain [9]. Accompanying substrate reduction ranged from 0 to 80 % [9].

More consistent reduction in biochemical measures of storage ranging from 12 to 73 % was achieved in LSD models murine mucopolysaccharidosis (MPS) IIIA and canine MPS I, where high dose, frequent and sustained intra-CSF ERT was employed [10–13]. A milder neurologic phenotype is observed in these MPS models compared to canine fucosidosis, and MPS I dogs receiving monthly and quarterly intrathecal infusions also received ERT intravenously [13]. In MPS IIIA mice, greater substrate reduction was achieved in the midbrain and cerebellum, close to the cerebromedullary infusion site [11]. The response of brain lesions to ERT in MPS IIIA mice was variable. Immunohistochemical markers of substrate accumulation demonstrated reductions, but inflammatory lesions and axonal dystrophy persisted in the inferior colliculus, hippocampus and thalamus [11]. Lysosomal expansion, axonal spheroids, hypomyelination, increased apoptosis and gliosis all require repair in fucosidosis, and the neuropathological response to low-dose, monthly intracisternal infusion of same-species recombinant enzyme in canine fucosidosis has yet to be reported.

In this study, we further evaluated the efficacy of reduced substrate storage after intra-CSF delivery of canine α-L-fucosidase in our previously reported cohort [9], using quantitative techniques to evaluate neuroinflammatory, myelin, lysosomal and neuronal pathology in preclinical fucosidosis.

## Methods

### Intracisternal injection experiments and sample collection

Monthly intracisternal infusions of recombinant canine α-L-fucosidase were administered in fucosidosis-affected English Springer spaniel pups (*n* = 3, affected enzyme treated (AET) 1, AET2, AET3) from 8 weeks of age for 3 months. Phosphate buffered saline (PBS) vehicle infusions were performed in age-matched fucosidosis-affected controls (*n* = 3, affected vehicle treated (AVT)1, AVT2, AVT3) and unaffected controls (*n* = 3, control vehicle treated (CVT)1, CVT2, CVT3). Biochemical data from this cohort of dogs is reported in [9], where detailed description of the intracisternal infusion procedure, and manufacture of replacement enzyme can be found. Briefly, 6.9U (0.96 mg/dose) of enzyme in 150–204 μL of PBS for AET dogs or 150 μL PBS vehicle for AVT and CVT animals was slowly infused via cisternomedullary CSF cannula under anesthesia over a period of 2 min. Dog AVT2 was euthanized at 12 weeks following an adverse anesthetic response. The remaining dogs were euthanized at 16 weeks, 48 h after the third infusion with an intravenous overdose of phenobarbital sodium at 150 mg/kg. Neural tissues were collected for gene expression and histological analysis. All procedures were approved by the Animal Ethics Committee at Westmead Hospital, The University of Sydney and followed the Australian Code of Practice for the Care and Use of Animals for Scientific Purposes.

### Histology and immunohistochemistry

Superficial frontal cortex (0.2 mm depth from the meningeal surface), deep frontal cortex (2–4 mm depth), corpus callosum, striatum, medulla, molecular layer of the cerebellum, cerebellar nuclei and cervical spinal cord were fixed in 10 % neutral buffered formalin for paraffin, or 2.5 % glutaraldehyde for epoxy resin embedding. Paraffin embedded sections were cut at 5 μm and placed on plain or silane coated slides prior to histochemical or immunohistochemical staining. Resin semi-thin sections were cut at 0.5–1 μm thickness and stained with 1 % toluidine blue. Histochemical stains luxol fast blue with periodic acidic Schiff (LFB/PAS) and haematoxylin and eosin (H&E). Immunohistochemical staining using glial fibrillary acid protein for astrocytes (GFAP, 1:2000, polyclonal rabbit anti-GFAP, Dako, Glostrup, Denmark), ubiquitin for axonal spheroids (1:4000, polyclonal rabbit anti-ubiquitin, Dako), and *Ricinus communis* agglutinin-1 lectin for microglia (RCA-1, 1:2500, biotinylated, Vector Laboratories, Burlingame, CA, USA) were performed as described in [3]. Caspase-6 for apoptosis (CASP6, 1:2000, polyclonal rabbit anti-CASP6, Abcam, Cambridge, UK) and 2’,3’-cyclic-nucleotide 3’-phosphodiesterase for terminal differentiating mature oligodendrocytes (CNPase, 1:400, monoclonal mouse anti-CNPase, Millipore, Temecula, CA, USA) was performed as described in [4]. All immunostaining was visualized using 3,3’-diamenobenzidine substrate and counterstained in hematoxylin.

### Quantitative assessment of neuropathology

All quantification was undertaken with observers blind to the identity of the sample. Matched regions were quantified from each dog as follows: lateral lobe of frontal cortex superficial layers 1–4, and deep layers 5 and 6, corpus callosum, striatum (globus pallidus), thalamus, medulla and cervical spinal cord.

#### Astrocytosis, microgliosis, hypomyelination and vacuolation

A consistent block design was used to select 15 fields in the defined neuroanatomical regions using the Leica Q500MC image processing and analysis system (Leica Cambridge Ltd, Cambridge, UK).

To quantify ‘astrocytosis’, ‘microgliosis’, and ‘myelin loss’ the percentage of positive staining for GFAP, RCA-1 and LFB/PAS (blue) tissue respectively, was averaged from 15 high-power fields as described previously [2, 3].

‘Vacuolation’ or the percentage of area vacuolated was determined by measuring the area occupied by H&E stain and subtracting the total area to give the transparent unstained area, as described in [2].

Photomicrographs of GFAP, H&E, LFB/PAS and RCA-1 stained sections were captured using a digital camera (Olympus DP-70, Olympus Australia Ltd, Sydney, Australia) attached to a light microscope (Olympus BX-51, Olympus Australia Ltd).

#### CASP6 apoptosis and oligodendrocyte counts

CASP6 staining and CNPase positive oligodendrocyte counts were quantitatively assessed in photomicrographs captured using a digital camera (Olympus DP-70, Olympus Australia Ltd) attached to a light microscope (Olympus BX-51, Olympus Australia Ltd) and functions available in ImageJ (National Institutes of Health, USA). Percentage of CASP6 staining in the cerebellar white matter and corpus callosum was measured in 10–15 fields of view captured at 400× magnification using the automated threshold function following pre-processing to the hue, saturation and brightness color space. To assess the number of mature oligodendrocytes, manual counts using the ‘cell count’ tool were performed on 10–15 fields of view of the corpus callosum and cerebellar white matter captured at 1000× magnification. Oligodendrocytes were identified as cell nuclei with CNPase positive cytoplasmic staining. Counts were expressed as total number of oligodendrocytes per mm^2^ cerebral or cerebellar white matter. Manual counts of CASP6 positive Purkinje cells were performed on 15 fields of view captured at 400× magnification. The total number of Purkinje cells and the number of CASP6 positive Purkinje cells at the border of the molecular and granule cell layers were counted. Counts were expressed as the percentage of positive CASP6 Purkinje cells per 1000 μm length of the border. The absence or presence of vacuolation and degenerate Purkinje cells with baskets were also noted.

#### Pyramidal neuron counts, perivascular spaces and lysosomal expansion within neurons

Pyramidal neuron counts and measurement of perivascular spaces were performed as described in [3]. For both measurements, images were collected at high power. The number of pyramidal neurons present in the fusiform layer of the frontal cerebral cortex were counted and expressed per 100000 μm^2^. Perivascular space measurements corresponding to the Virchow-Robins space were taken at peri-arteriole and peri-venule regions within the parenchyma of the dorsal frontal cortex, within 1–2 cm of the mid-sagittal sulcus. The average perivascular space was measured by determining the width of the space between the endothelial membrane and the adjacent parenchyma. A minimum of 15 perivascular spaces were measured per dog.

To assess the lysosomal expansion within neurons ‘vacuoles per neuron’ were counted in photomicrographs of semi-thin resin sections of the superficial (layers 1–4) and deep (layers 5 and 6) frontal cortex captured at 1000× magnification. In each neuroanatomically defined region, 15 complete neurons (distinct cytoplasm and nucleus) were identified, and the number of membrane-bound vesicles identified as putative lysosomes within neuronal cytoplasm were manually counted and averaged. Criteria for positively identifying neurons were large nucleus, clumped chromatin, large nucleoli, cytoplasm with Nissl substance (ribosomes, rough endoplasmic reticulum), distinctive axonal processes and adjacent supporting glia.

### Gene expression of neuroinflammatory and lysosomal membrane protein markers

To assess the value of proinflammatory cytokines and lysosomal membrane proteins as markers of reduced storage and secondary inflammation following treatment, relative expression of interleukin (IL)-6 and −8, transforming growth factor-β (TGFβ) and lysosomal membrane associated protein-I (LAMP1) was determined using quantitative reverse transcriptase polymerase chain reaction (qRT-PCR). Following necropsy, the lateral lobe of the frontal cerebral cortex from each of the AET, AVT and CVT dogs was snap frozen in liquid nitrogen at necropsy prior to RNA extraction (RNeasy Lipid Tissue Mini kit, QIAGEN, MD, USA). cDNA was synthesized from 2 μg of total RNA using reverse transcriptase (SuperScript III, Life Technologies, CA, USA) and oligo(dT) primer (Life Technologies). PCR primers (Additional file 1) were derived from the literature (GAPDH [14]) or designed from canine-specific cDNA sequences (GenBank/EMBL data bank, CanFam 2.0 May 2005 annotation) using Primer3 [15]. Primer stocks were diluted to a working concentration of 100 ng/μL. All primers were tested for PCR functionality by running reaction products on 2 % agarose gels in tris-acetate-EDTA.

qRT-PCR was performed in triplicate 20 μL reactions using a Rotor-Gene 6000 (QIAGEN) under the following conditions: initial denaturation at 95 °C for 5 min, followed by 35 cycles of 95 °C for 30 s, 60 °C for 30 s, 72 °C for 30 s in the presence of 3 mM MgCl_2_ and 0.8 μL of 1X SYBR® Green I (Molecular Probes, Eugene, OR, USA). A melt run was performed at 72–95 °C to confirm a single product. Relative gene expression of was performed by normalizing to GAPDH, as an internal standard.

### Statistical analysis and data representation

Significant differences between groups were determined by ANOVA (GenStat 11th Edition, VSN International Ltd, Hertfordshire, UK). All data were analyzed using logit transformation due to unequal variances. *p* < 0.05 was regarded as significant, *p* < 0.001 as highly significant. As AVT2 was 12 weeks, and AVT1 and 3 were 16 weeks at necropsy data were analyzed with and without AVT2 and differences noted in results.

**Fig. 1:**
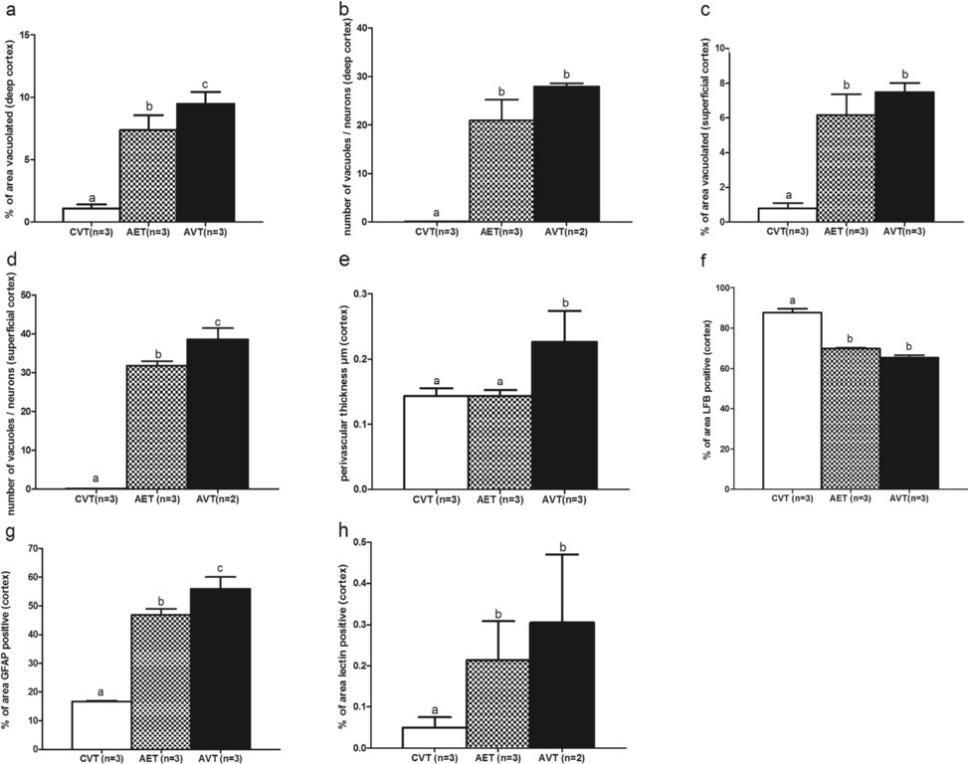
Quantification of neurodegenerative and inflammatory markers in frontal cerebral cortex following intracisternal enzyme infusion. Quantification of (**a**) area vacuolation in deep frontal cerebral cortex, (**b**) ‘vacuoles per neuron’ in deep frontal cerebral cortex, (**c**) vacuolation in superificial frontal cerebral cortex, (**d**) ‘vacuoles per neuron’ in deep frontal cerebral cortex, (**e**) average thickness of perivascular space in frontal cerebral cortex, (**f**) LFB/PAS staining in deep white matter tracts of frontal cerebral cortex following enzyme infusion, (**g**) area GFAP staining in frontal cerebral cortex, (**h**) area RCA-1 lectin staining in frontal cerebral cortex. Different letters (^a, b, c^) indicate significant difference between groups at *p* < 0.05

A correction index (CI) was developed as a measure of the extent of correction achieved by ERT in AET animals relative to AVT controls for vacuolation, substrate and additional neurodegenerative markers. CI was calculated as the mean measure in the AVT group minus the mean measure in the AET group divided by the mean measure in the AVT group, eg. in Fig. 1a (9.38–7.17)/9.38 = 0.24 CI. A large absolute value close to 1 indicates a large change in that maker in AET animals compared to AVT controls.

## Results

All dogs tolerated the infusion procedure well, and there was no histological evidence of reactive meningitis in either of the infusion vehicle treated AVT and CVT dogs, or the AET recombinant canine α-L-fucosidase treated animals. Fucosidosis lesions including vacuolation (lysosomal expansion), hypomyelination, astrocytosis, microgliosis and neuron loss were ameliorated to varying degrees in AET brain. There were small, consistent reductions in vacuolation as indicated by the CI across forebrain regions of AET animals compared to AVT controls (Table 1). This included the corpus callosum, striatum and thalamus. Small reductions in vacuolation were also observed in hindbrain regions and spinal cord (Table 1). Axonal spheroid formation in AET animals remained unchanged in all regions of the brain.

**Table 1:** Percentage of vacuolation and CI of deep regions of cerebrum

Region	CVT (*n* = 3)	AET (*n* = 3)	AVT (*n* = 3)	CI
Corpus callosum	0.55 ± 0.1 ^a^	5.78 ± 1.1 ^b^	6.66 ± 1.4 ^b^	0.13
Striatum	0.31 ± 0.1 ^a^	2.65 ± 1.0 ^b^	3.52 ± 1.3 ^b^	0.25
Thalamus	0.34 ± 0.1 ^a^	3.07 ± 0.9 ^b^	4.03 ± 1.3 ^b^	0.23
Medulla	0.78 ± 0.25 ^a^	7.65 ± 2.4 ^b^	7.68 ± 2.2 ^b^	0.03
Spinal cord	4.63 ± 1.5 ^a^	10.78 ± 3.5 ^b^	12.79 ± 4.1 ^b^	0.16

Mean ± SEM. ^a,b^ different letters indicate significant difference between affected dogs and unaffected controls at *p* < 0.05

### Frontal cerebral cortex

#### Lysosomal expansion in superficial and deep cerebral cortex

**Fig. 2:**
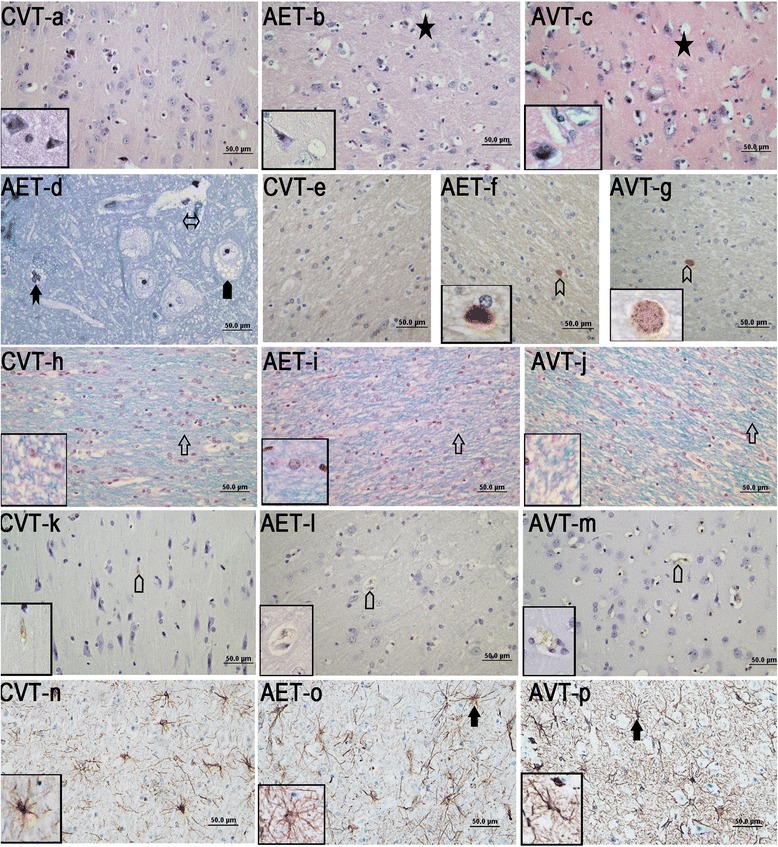
Histology and immunostaining in the frontal cerebral cortex of CVT, AET and AVT dogs. **a**–**c** H&E sections of CVT, AET and AVT showing vacuolation, (**d**) toluidine blue section of AET showing vacuoles in different cell types, (**e**–**g**) ubiquitin immunostaining, (**h**–**j**) LFB/PAS staining of deep white matter tracts of frontal cerebral cortex, (k-m) RCA-1 lectin (k-m) and GFAP (**n**–**p**) immunostaining staining in frontal cerebral cortex of CVT, AET and AVT.  indicate vacuolation,  putative microglia with numerous membrane bound vesicles,  membrane bound vesicles in neuron,  indicate enlarged perivascular space,  indicate LFB staining, indicate ubiqutin positive deposits,  indicate lectin (RCA-1) staining and indicate GFAP stained astrocytes. Inserts are at 1000× magnification

Mean vacuolation in AET dogs was significantly reduced in cortical layers 5 and 6 compared to untreated AVT animals (Fig. 1a), in which spongy vacuolation of the deep cortical parenchyma is prominent. In cortical layers 1–4, there was no significant change in the percentage of vacuolation with administration of intracisternal ERT (Fig. 1c). However, quantitative assessment of lysosomal expansion within neurons using ultrastructual examination demonstrated a significant decrease in the number of ‘vacuoles per neuron’ in the superficial cortex (0.18 CI) of AET dogs (Fig. 1b). There was no significant change (0.21 CI) in ‘vacuoles per neuron’ identified between AET and AVT dogs in cortical layers 5 and 6, where despite reduction in overall mean vacuolation, degenerative changes to neurons persisted. These included cytoplasmic distortion, disintegrated Nissl substance and chromatin condensation (Fig. 2b).

#### Perivascular regions

Measurement of Virchow-Robins spaces within the pyramidal neuronal layer of the frontal cortex revealed that their thickeness in AET brain were significantly reduced compared to AVT dogs, ranging from 0.12 to 0.16 μm, similar to the mean of 0.14 μm observed in unaffected controls (0.39, Fig. 1e). Accumulation of vacuolated microglia and macrophages around vascular regions causing distention of perivascular spaces was prevalent in AVT frontal cortex.

#### Hypomyelination

Following intracisternal ERT, the number of oligodendrocytes per mm^2^ in the corpus callosum was significantly preserved compared to untreated AVT dogs (Table 2, Fig. 4a-c). Despite this, there was no significant change in the density of cortical white matter tracts observed with LFB/PAS staining in AET dogs compared to AVT (0.07CI, Fig. 1f).

**Table 2:** The effect of intracisternal ERT on measures of oligodendrocyte loss and apoptosis

Measure	CVT (*n* = 3)	AET (*n* = 3)	AVT(*n* = 3)	CI
Oligodendrocytes per mm^2^ white matter				
Cerebellar white matter	907 ± 66 ^a^	584 ± 66 ^b^	521 ± 66 ^b^	−0.12
Corpus callosum	1366 ± 60 ^a^	889 ± 60 ^b^	603 ± 60 ^c^	−0.47
Percentage of CASP6 staining				
Cerebellar white matter	1.2 ± 0.5 %	1.3 ± 0.5 %	2.0 ± 0.8 %	0.35
Corpus callosum’	0.5 ± 0.2 % ^a^	5.0 ± 1.9 % ^b^	5.5 ± 2.2 % ^b^	0.09
Purkinje cells	20.9 ± 7.2 % ^a^	49.2 ± 7.2 % ^b^	^c^ 77.7 ± 7.2 % ^c^	0.37

Mean ± SEM. ^a,b,c^ different letters indicate significant difference at *p* < 0.05

#### Astrocytosis and microgliosis

Following intracisternal ERT, astrocytic processes appeared reduced in length (Fig. 2p–r), and in the cerebral cortex GFAP staining was significantly reduced in AET dogs (0.16 CI, Fig. 1g). There was no change in thalamic astrocytosis following enzyme infusion (AET: 36.6 %, AVT: 36.9 %, 0.008 CI).

In the frontal cerebral cortex, there was a trend towards reduced microgliosis in AET animals (0.35CI, Fig. 1h) with fewer RCA-1 lectin positive microglia (Fig. 2m–o). In both AET and AVT animals, activated microglia and macrophages were localized adjacent to intense neuronal and endothelial vacuolation.

#### Axonal spheroids, pyramidal neuron loss and apoptosis

**Fig. 3:**
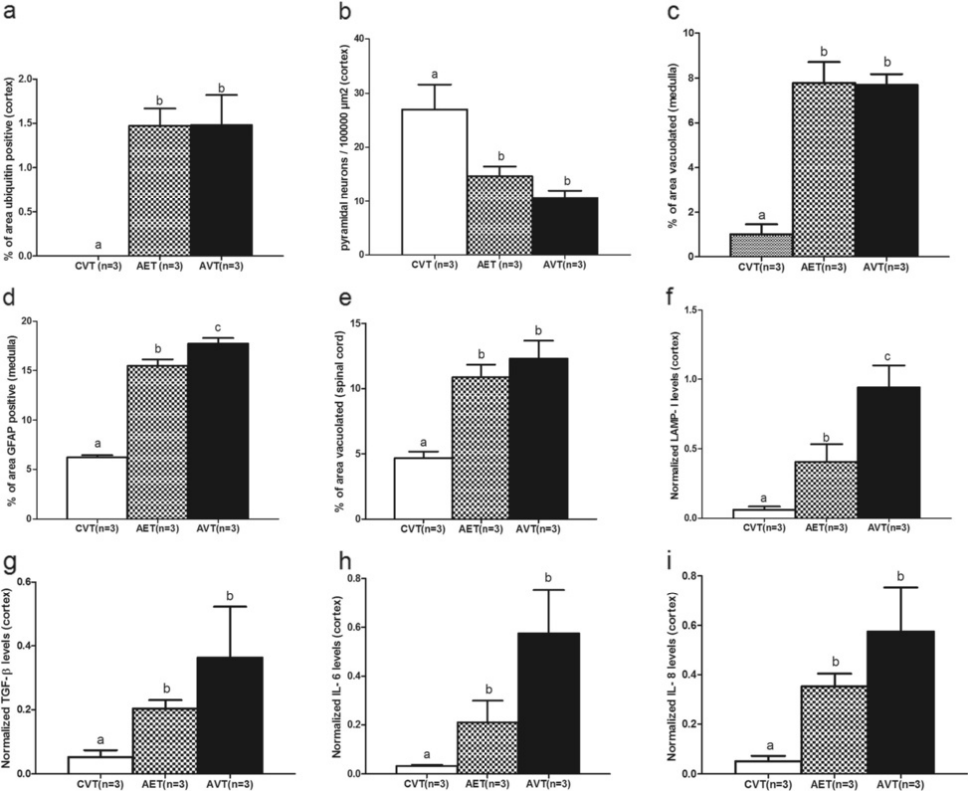
Quantification of neurodegenerative and inflammatory markers in CNS following intracisternal enzyme infusion. Quantification of (**a**) area of ubiquitin staining in frontal cerebral cortex, (**b**) pyramidal neurons in frontal cerebral cortex, (**c**) area vacuolation and, (**d**) area of GFAP staining in medulla, (**e**) area vacuolation in cervical spinal cord grey matter. Expression levels of (**f**) LAMP-1, (g) TGF-β, (**h**) IL-6 and (**i**) IL-8 genes in superficial frontal cerebral cortex. Different letters (^a, b, c^) indicate significant difference between groups at *p* < 0.05

**Fig. 4:**
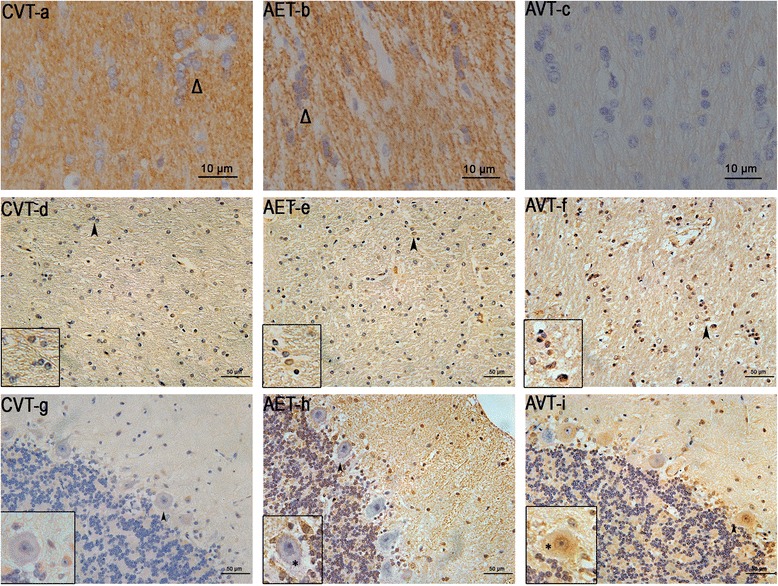
Oligodendrocytes and apoptosis in the corpus callosum and cerebellum following intracisternal enzyme infusion. CNPase immunostaining in the corpus callosum (**a**–**c**) and CASP6 immunostaining in the corpus callosum (**d**–**f**) and cerebellum (**g**–**i**) of CVT, AET and AVT. Δ indicates CNPase positive oligodendrocytes. Inserts (**d**–**f**) are at 1000× magnification (arrowheads)

Axonal spheroid formation in the corpus callosum (Fig. 2j–l) did not change with enzyme infusion (Fig. 3a). Pyramidal neuron loss was less severe in AET frontal cerebral cortex compared to AVT, but there was no significant improvement (0.22 CI, Fig. 3b). CASP6 staining remained elevated in AET corpus callosum compared with CVT controls, with no effect of intracisternal ERT (Fig. 4d–f).

### Cerebellum

Vacuolation, astrocytosis and hypomyelination demonstrated minor reductions in the cerebellum following intracisternal ERT, but these did not reach significance. Lower levels of CASP6 staining was present in the cerebellar white matter of AET dogs compared to AVT (0.35 CI), but cerebellar oligodendrocyte numbers remained unchanged despite intracisternal ERT (Table 2).

The number of Purkinje cells positive for apoptosis vulnerability marker CASP6 were significantly decreased in AET compared to AVT (0.37 CI), but remained elevated compared to CVT animals (Table 2, Fig. 4g–i). All CASP6-positive Purkinje cells in AET and AVT animals demonstrated vacuolation, and the occasional degenerate empty basket, as well as CASP6-positive Bergmann glia were present (Fig. 4g–i). There was no difference in the total number of Purkinje cells observed between AET and AVT (Table 3).

**Table 3:** Neurodegenerative markers in cerebellum following enzyme replacement therapy

Neurodegenerative markers in cerebellum	CVT (*n* = 3)	AET (*n* = 3)	AVT (*n* = 2–3)	CI
Vacuolation in molecular layer	0.26 ± 0.0 ^a^	5.47 ± 1.1 ^b^	5.52 ± 1.2 ^b^	0.09
Vacuolation in cerebellar nuclei	0.52 ± 0.2 ^a^	3.38 ± 1.6 ^b^	4.87 ± 2.3 ^b^	0.30
Purkinje cell counts	12 ± 0.8 ^a^	10 ± 0.5 ^b^	10 ± 0.2 ^b^	0.00
Myelination	67.18 ± 0.8 ^a^	58.00 ± 3.2 ^b^	55.02 ± 2.3 ^b^	0.05
Astrocytosis	6.77 ± 0.4 ^a^	13.05 ± 0.8 ^b^	14.24 ± 0.9 ^b^	0.08
Axonal spheroids	0.01 ± 0.5 ^a^	0.84 ± 0.6 ^b^	0.84 ± 0.0* ^b^	0.00

Mean ± SEM. ^a,b^ different letters indicate significant difference between affected dogs and unaffected controls at *p* < 0.05

Axonal spheroids present in the cerebellar white matter did not response to enzyme infusion (Table 3).

### Medulla and spinal cord

The percentage of vacuolation in the medulla was unchanged by intracisternal ERT (Fig. 3c). Enzyme infusion led to a small but highly significant reduction in GFAP staining in AET medulla (0.13 CI, Fig. 3d). In the cervical spinal cord there was a trend towards reduced vacuolation (0.16 CI, Fig. 3e).

### LAMP-1 and inflammatory gene expression in fucosidosis brain following ERT

Gene expression (Fig. 3f–i) demonstrated positive responses to therapy. LAMP-1 expression in the superficial frontal cortex was significantly reduced following enzyme infusion (0.57 CI, Fig. 3f). TGFβ gene expression showed decreases with intracisternal ERT (0.44 CI, Fig. 3g) and both IL6 and IL8 demonstrated a trend towards reduction in AET cerebral cortex compared to AVT (0.39 CI, Fig. 3i).

## Discussion

This study provides clear evidence of differential corrective effects in brain and spinal cord following intracisternal delivery of replacement enzyme in LSD with severe neurological involvement. It is the first study to identify relationships between reduced storage measured by vacuolation, and a broad range of other neurodegenerative lesions following partial enzyme replacement and substrate correction in fucosidosis. Administration of species-matched recombinant enzyme was safe, and well-tolerated by the dogs in this study, with no histological evidence of reactive meningitis to either PBS infusion vehicle or the treatment enzyme following repeated injection. This was supported by previous findings of normal cellularity and protein levels in CSF, and no antibody response to the recombinant enzyme or infusion vehicle for all treatment groups [9]. Despite persistence of small vacuoles in endothelial cells at the ultrastructural level, decreases in the perivascular accumulation of macrophages in the cortex was the most responsive marker to treatment, and is consistent with perivascular uptake of enzyme infused via CSF [16]. The small reductions in measures of vacuolation, astrocytosis, microgliosis, ‘vacuoles per neuron’, pyramidal neuron counts and hypomyelination in fucosidosis-affected cerebral cortex, cerebellum, medulla and spinal cord are consistent with studies in MPS I and IIIA dogs, which have demonstrated that these regions are responsive to biochemical correction [10, 17]. Axonal dystrophy and Purkinje cell loss showed no response, but reductions in the number of CASP6-positive Purkinje cells suggest that rescue of Purkinje neurons by intracisternal ERT may be seen over time with continued treatment.

Infusion of enzyme via CSF contrasts with HCT, where enzyme is delivered by myeloid derived donor cells migrating diffusely from circulation to populate the brain throughout life, forming at least 10 % of all ‘resident’ microglia [18]. Monthly intracisternal infusions provide a transient source of replacement enzyme, and this likely contributed to the subtle and variable effects observed in intracisternal-ERT treated animals. Measures strongly associated with substrate accumulation including cerebrocortical vacuolation, ‘vacuoles per neuron’, perivascular accumulation and LAMP-1 gene expression demonstrated significant reductions in fucosidosis dogs treated with intracisternal ERT. This aligns with the moderate and consistent biochemical correction achieved in the same cohort of animals. Levels of fucosylated oligosaccharide were reduced by 23–80 % in all regions of the CNS sampled, except the thalamus and corpus callosum [9]. Secondary lesions, other than cortical astrocytosis, were refractory to this regime of intracisternal ERT although there was a trend towards reduced disease burden across all CNS regions sampled. In the future, an increased cohort of animals, or additional markers including fucose-specific lectins [19], or immunohistochemistry against the recombinant infusion enzyme may enable more precise evaluation of the distribution and regional effects of the ERT infusion.

The variability in the region and the extent of neuropathological improvement likely relates to both the pattern of enzyme absorption from the CSF into the parenchyma of the brain, and the severity of pre-existing lesions. Studies in canine MPS IIIA demonstrate that ERT delivered via CSF follows perivascular spaces, with subsequent centrifugal spread to neurons, astrocytes and oligodendroglia [16]. This was determined using immunohistochemistry against recombinant N-sulfoglucosamine sulfohydrolase used in these ERT studies [16]. The significant reduction in the thickness of Virchow-Robins spaces with intracisternal-ERT treated in fucosidosis dogs towards that seen in unaffected controls, supports this. However, lesions in the cerebellum, the structure closest to the infusion site of the cisterna magna, demonstrated a poor response to therapy. This is difficult to reconcile, as the cerebellum had high levels of enzyme activity 48 h after the final infusion (25–36 %) and demonstrated 23–45 % reduction in oligosaccharide accumulation [9].

High enzyme activity in the cerebellum is consistent with studies of CSF dispersal following intracisternal infusion in canines, where the pulsating flow of CSF through the subarachnoid space of the CNS initially drives infusions rostrally towards the cerebrum and caudally towards the cervical spinal cord, allowing diffusion into the cerebellum [20]. However, poor neuropathological responses to therapy in the cerebellum have also been identified in MPS I and IIIB dogs receiving enzyme replacement through gene therapy. Again, this is a treatment method that provides long-lasting enzyme replacement, and in these animals significant levels of correction in cortical and thalamic vacuolation were achieved, without correction of lysosomal expansion in the cerebellum [17]. It is possible that despite the 23–45 % biochemical reduction in oligosaccharide achieved in the cerebellum by α-L-fucosidase infusion, pre-existing lesions were unable to be reversed, and this is reflected in the slight reductions observed in cerebellar vacuolation and astrocytosis.

Despite this, infusion of α-L-fucosidase slowed the continued development of neurodegenerative lesions, including secondary neuroinflammatory changes. Cerebrocortical astrocytosis demonstrated a significant response to intracisternal ERT, and cortical upregulation of cytokine genes, and RCA-1 lectin stained microgliosis in showed subtle reductions. Expression of IL6 and IL8 genes were also reduced with treatment and these cytokines may have potential as markers of storage-induced inflammation which may be monitored in blood or CSF. Additional inflammatory genes including major histocompability class II, chemokine C-C motif receptor-1 and 5, cathepsin S and IL1A are upregulated in preclinical fucosidosis frontal cortex [21] and may also be suitable candidates for monitoring responses to therapy in the future.

Although, the 6.9U monthly dose for 3 months was insufficient to halt the inflammatory changes in canine fucosidosis, minimizing the extent of neuroinflammation may be integral to providing a beneficial clinical outcome. Chronic neuroinflammation is known to mediate apoptosis [22], the ultimate cause of clinical dysfunction. Mild and regionally variable reductions in inflammatory pathology in fucosidosis following intracisternal ERT are consistent with findings of regional variable reductions of lectin-stained microglia in MPS IIIA mice, which received ERT via CSF [11]. The decreasing inflammatory responses observed with reductions in cerebrocortical astrocytosis and microgliosis in fucosidosis is likely a downstream effect of reduced substrate accumulation and lysosomal expansion demonstrated in layers 5 and 6. It also corresponded with significantly reduced LAMP-1 gene expression, which as with other lysosomal membrane proteins is a sensitive marker of lysosomal system expansion in LSD [10, 11].

Axonal dystrophy (spheroids) was resistant to intracisternal ERT in fucosidosis, as it has been resistant to intracisternal ERT in MPS IIIA mice [11], and early haematopoietic chimerism and reversal of substrate accumulation in ovine and bovine α-mannosidosis [23, 24]. The development of axonal dystrophy coincided with the period of treatment since axonal spheroids were rare in the cortex and cerebellum at 8 weeks in canine fucosidosis [3], and widespread by 16 weeks. This indicates that partial enzyme replacement is insufficient to arrest the abnormality in axonal transport. Some reduction of axonal spheroids were observed in the frontal cortex of α-mannosidosis cats treated with HSCT at 10 and 12 weeks of age compared to 9 week old untreated [25], but overall axonal dystrophy appears to be resistant to all enzyme targeted therapies for LSD.

In contrast, myelin abnormalities (both demyelination and hypomyelination) demonstrate positive responses to enzyme target therapies in LSD, although reductions in fucosidosis hypomyelination were mild in the current study. Genes encoding myelin-specific proteins 2’,3’-cyclic nucleotide 3’-phosphodiesterase (CNP), oligodendrocyte myelin paranodal inner loop protein (OPALIN), myelin-associated glycoprotein (MAG), and myelin and lymphocyte (MAL) were significantly downregulated in preclinical fucosidosis-affected cortex [21], consistent with a 56 % loss of oligodendrocytes [4] and the decline in the density of LFB/PAS stained tracts. Hypomyelination was slightly ameliorated following intracisternal ERT in preclinical fucosidosis-affected dogs, and there was a 21 % increase in the survival of oligodendrocytes at 16 weeks of age. An improvement in brain myelination detected by magnetic resonance imaging (MRI) was reported in α-mannosidosis cats following gene therapy [26]. Hypomyelination was found to be improved in human fucosidosis HCT in MRI studies [7, 8], with a 4 year follow up showing that the treated child reached age appropriate levels of myelination [7]. Preservation of callosal oligodendrocytes in canine fucosidosis treated with intracisternal ERT supports the idea that the initial hypomyelinating deficits in this LSD are amenable to enzyme targeted therapy, and in the future monitoring myelin response to treatment by MRI may be a reliable and less invasive marker of improved brain environment and improved clinical outcomes.

The impact of therapy on neuronal survival and function is of the highest importance in averting irreversible mental retardation in LSD, but few studies have addressed the regional differences in neuron loss, the relationship to apoptosis or responsiveness to therapy. Neuron loss is widespread and progressive in canine fucosidosis with 0.9–1.2-fold decrease in cerebrocortical and cerebellar Purkinje neuron numbers, and increased apoptosis (deoxynucleotide transferase mediated dUTP nick-end labeling) at 16 weeks of age in both regions [3]. Early intracisternal ERT had a beneficial effect, reducing lysosomal expansion and neuron loss in the frontal cortex with significant decrease in the number of putative enlarged lysosomes within neurons and a trend towards reduced pyramidal neuronal loss. There were also positive reductions in cortical apoptosis with significantly fewer cells positive for CASP6, a marker of vulnerability to apoptosis.

The effect of intracisternal ERT on cerebellar neurons was less pronounced, with only a trend towards reduced apoptosis vulnerability in cerebellar white matter and no difference in total number of Purkinje cells present in treated and untreated fucosidosis cerebellum. This was despite a significant decrease in the number of Purkinje cells positive for CASP6. Reduced vulnerability to apoptosis likely does not translate to reduced Purkinje cell loss immediately, and the apparent regional difference in neuronal treatment response may relate to different levels of susceptibility in neuronal subpopulations to substrate accumulation. Continuation of this low-dose ERT regime in fucosidosis may be insufficient to prevent development of the cerebellar signs of ataxia, hypermetria and proprioceptive deficits which commence at 10–12 months of age. However, the increased survival of cortical pyramidal neurons with this treatment is promising, providing encouragement that ERT, like HCT, may delay the progressive cortical dysfunction if adequate enzyme levels are sustained from early life.

## Conclusions

Intracisternal ERT with species-matched recombinant enzyme was safe with no signs of reactive meningitis, and there were significant improvements in deep cerebrocortical vacuolation, superficial cortex ‘vacuole per neuron’, cortical astrocytosis, and cortical LAMP-1 gene expression in the fucosidosis CNS as a result of treatment. A consistent, albeit small response to treatment in other measures such as myelination, microgliosis and cortical neuron loss was established through quantification. Reduction of storage material in perivascular macrophages was the most responsive measure, consistent with greater exposure of perivascular regions to fluctuations in CSF-delivered enzyme activity. Trends towards improvement in almost all measures of neurodegeneration and inflammation in the frontal cortex, cerebellum, medullar and spinal cord provide promise that more intensive ERT would improve treatment outcomes. The importance of sufficient, sustained, very early treatment, before lesions establish and cause irreversible damage and clinical signs cannot be overstated in fucosidosis, and other lysosomal disease.

## Additional file

Additional file 1:
**Forward and reverse primer sequences with the amplicon size.** (PDF 103 kb)

## Abbreviations

AET: Affected enzyme treated
AVT: Affected vehicle treated
CASP6: Caspase-6
CI: Correction index
CNPase: 2’,3’-cyclic-nucleotide 3’-phosphodiesterase
CVT: Control vehicle treated
CSF: Cerebrospinal fluid
ERT: Enzyme replacement therapy
GFAP: Glial fibrillary acid protein
HCT: Haematopoietic cell transplantation
H&E: Haematoxylin and eosin
IL: Interleukin
LAMP1: Lysosomal membrane associated protein-I
LFB/PAS: Luxol fast blue with periodic acidic Schiff
LSD: Lysosomal storage disorder
MPS: Mucopolysaccharidosis
RCA-1: *Ricinus communis* agglutinin-1
TGFβ: Transforming growth factor-β
