# Supplementary material for: The effects of intracisternal enzyme replacement versus sham treatment on central neuropathology in preclinical canine fucosidosis
Source: Orphanet J Rare Dis. 2015 Nov 4;10:143. doi: 10.1186/s13023-015-0357-z (PMC4632352; doi:10.1186/s13023-015-0357-z)
Supplement: Additional file 1: — Forward and reverse primer sequences with the amplicon size. (PDF 103 kb) [file 13023_2015_357_MOESM1_ESM.pdf]

Forward and reverse primer sequences with the amplicon size.

| **Main primer** | **GenBank Accession No.** | **Sequence (5'-3')** | **Product size** |
|---|---|---|---|
| GAPDH Forward | **NM_001003142** | GCCAAAAGGGTCATCATCTC | |
| GAPDH Reverse | | GGGGCCATCCACAGTCTTCT | 228bp |
| LAMP-I Forward | **XM_003433089** | GCGAGCTATGAAACCAGGAG | |
| LAMP-I Reverse | | TGGGGAAAATCTGTGTGTCA | 232bp |
| IL-6 Forward | **XM_532488** | GGCTACTGCTTTCCCTACCC | |
| IL-6 Reverse | | TTTTCTGCCAGTGCCTCTTT | 198bp |
| IL-8 Forward | **XM_532409** | TCTTGGCAGCTTTTGTCCTT | |
| IL-8 Reverse | | GGGCCACTGTCAATCACTCT | 151bp |
| TGF-β Forward | **NM_001003309** | TTCCTGCTCCTCATGGCCAC | |
| TGF-β Reverse | | GCAGGAGCGCACGATCATGT | 393bp |
